# Supplementary figures and images for: Genetic Identification and Transcriptome Analysis of Lintless and Fuzzless Traits in Gossypium arboreum L
Source: Int J Mol Sci. 2020 Feb 29;21(5):1675. doi: 10.3390/ijms21051675 (PMC7084617; doi:10.3390/ijms21051675)

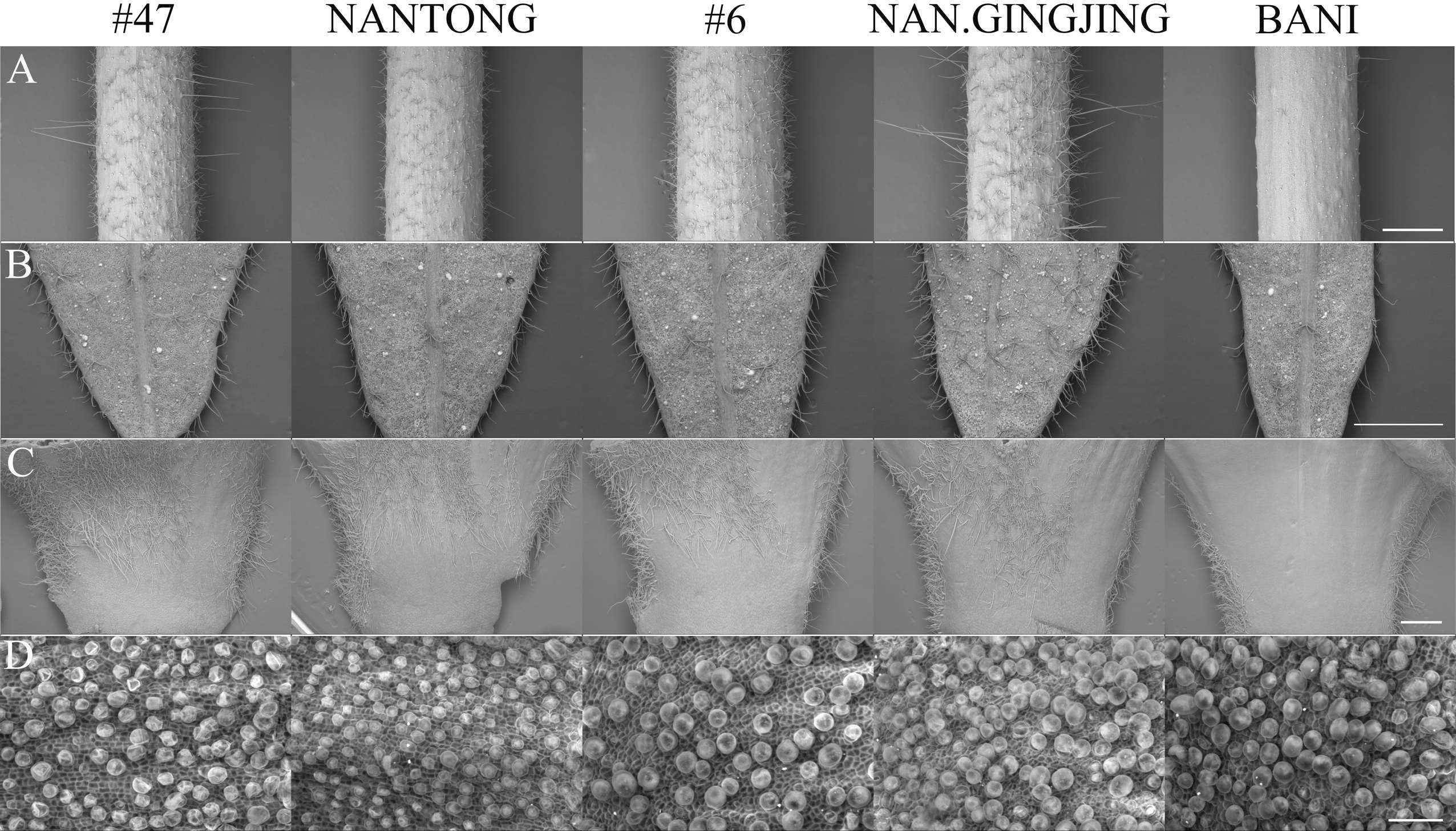

Supplement: Supplementary file 1 [file ijms-21-01675-s001.zip › figure S1.jpg]

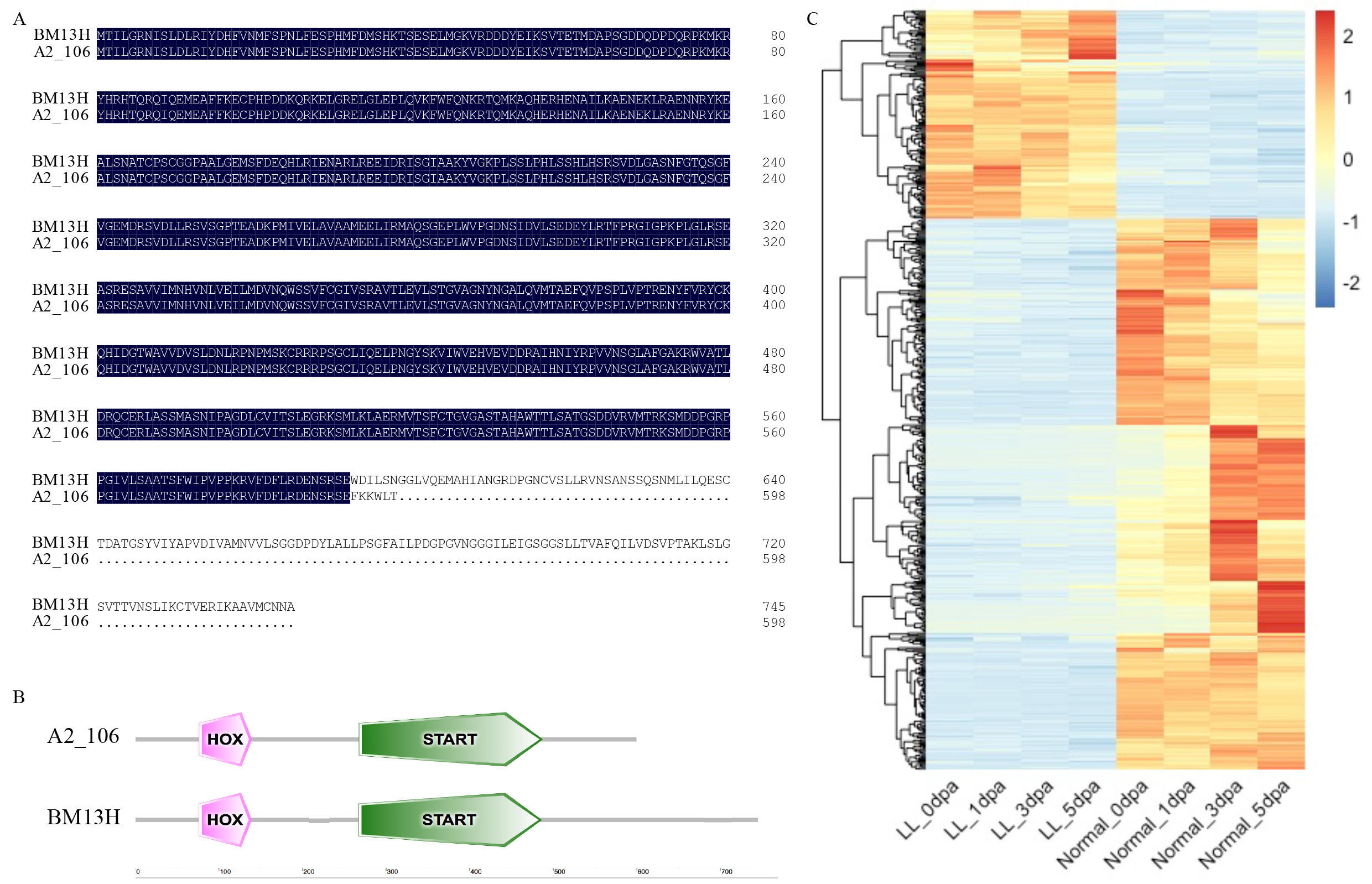

Supplement: Supplementary file 1 [file ijms-21-01675-s001.zip › figure S2.jpg]

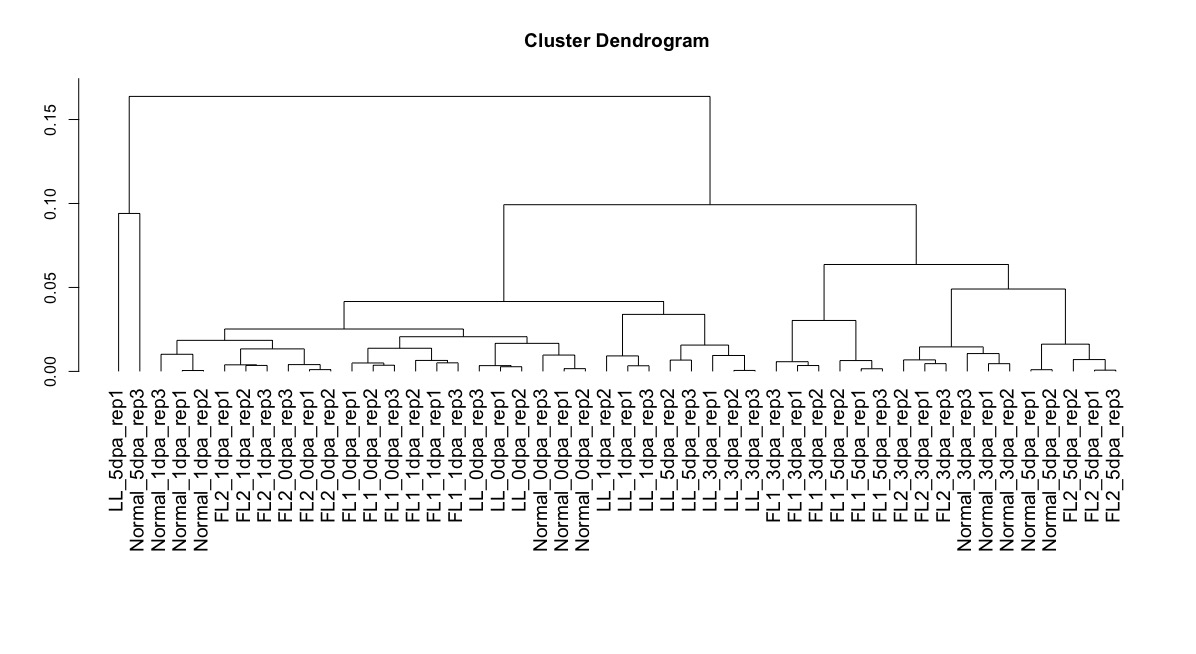

Supplement: Supplementary file 1 [file ijms-21-01675-s001.zip › figure S3.jpg]

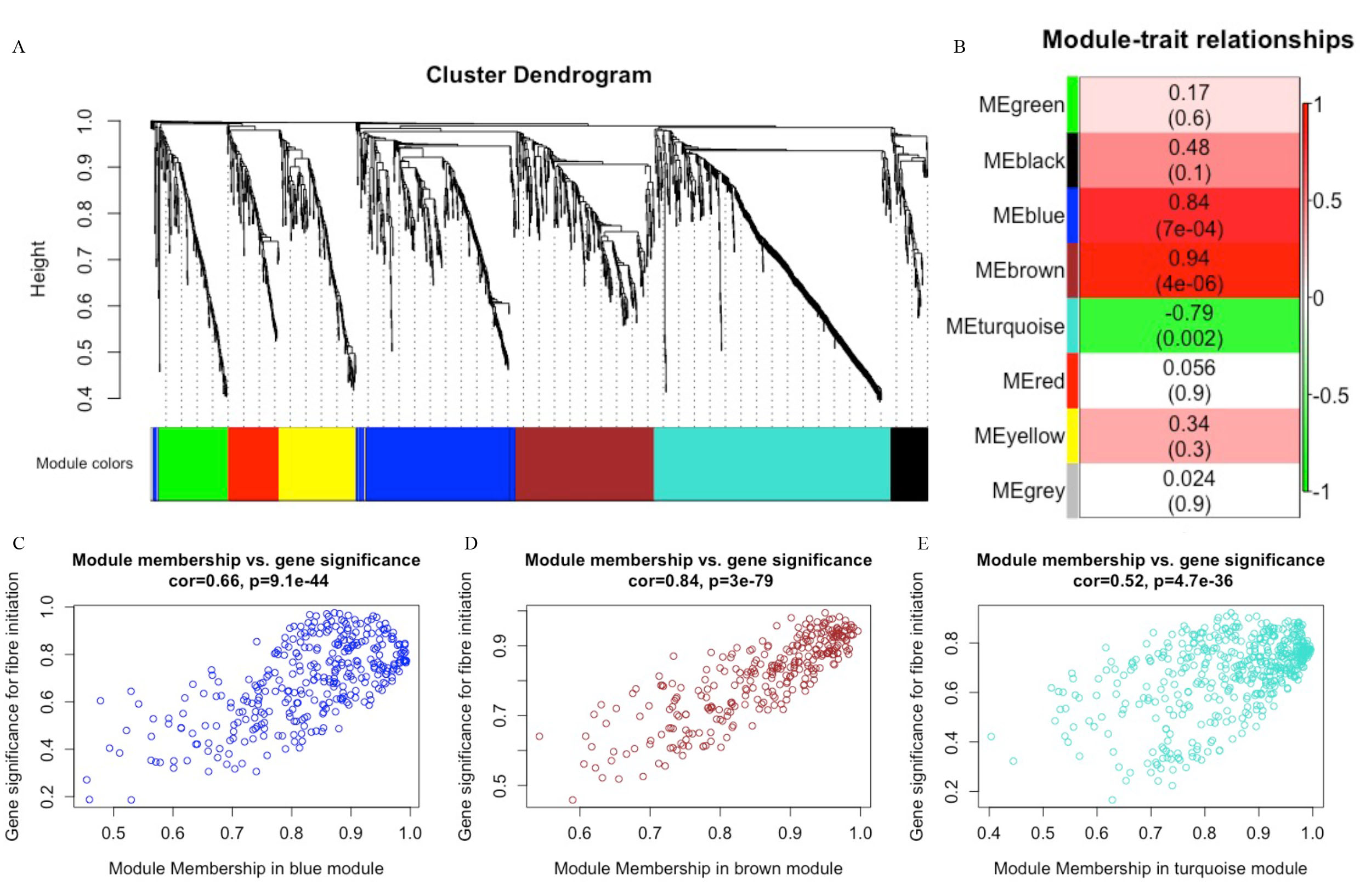

Supplement: Supplementary file 1 [file ijms-21-01675-s001.zip › figure S4.jpg]

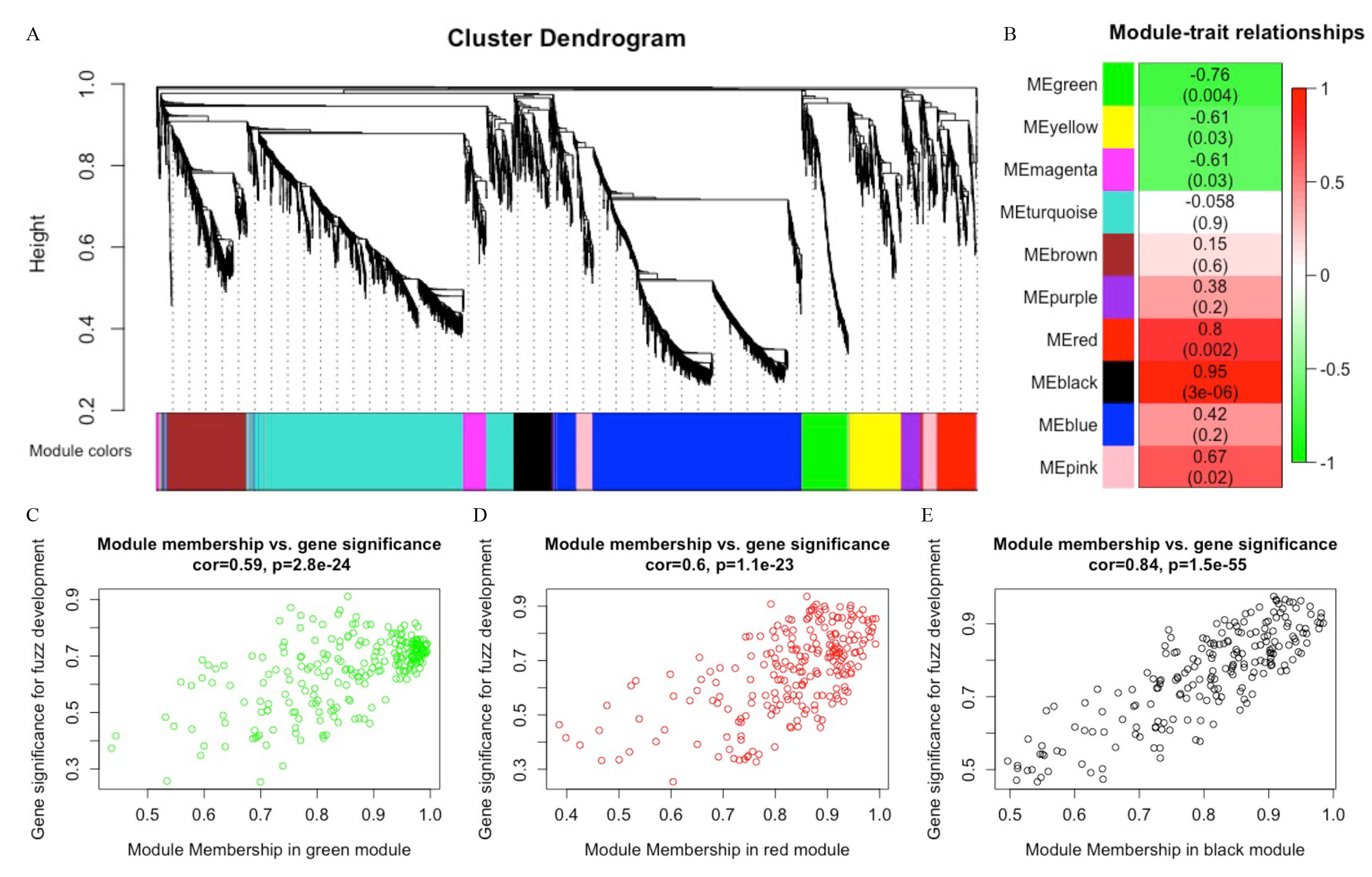

Supplement: Supplementary file 1 [file ijms-21-01675-s001.zip › figure S5.jpg]

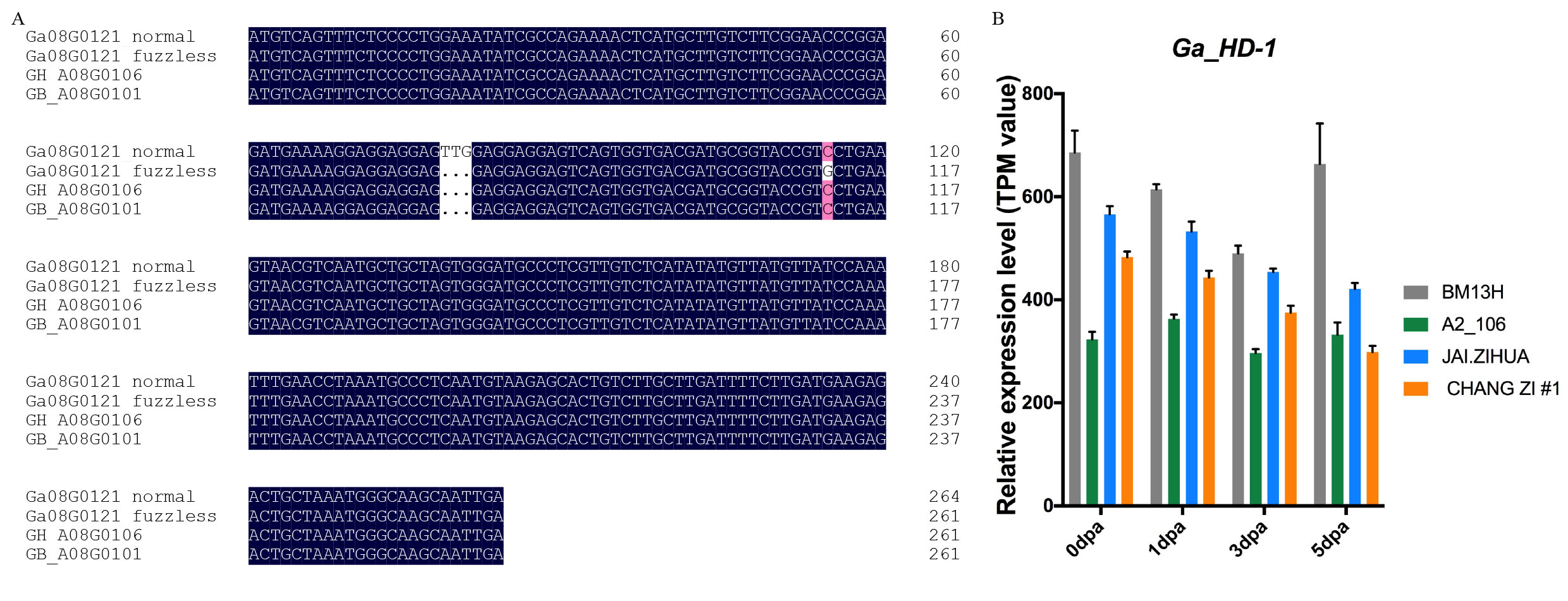

Supplement: Supplementary file 1 [file ijms-21-01675-s001.zip › figure S6.jpg]
